# Genetic and microbial determinants of azoxymethane-induced colorectal tumor susceptibility in Collaborative Cross mice and their implication in human cancer

**DOI:** 10.1080/19490976.2024.2341647

**Published:** 2024-04-24

**Authors:** Dan Li, Chenhan Zhong, Mengyuan Yang, Li He, Hang Chang, Ning Zhu, Susan E Celniker, David W Threadgill, Antoine M Snijders, Jian-Hua Mao, Ying Yuan

**Affiliations:** a Key Laboratory of Cancer Prevention and Intervention, China National Ministry of Education, Key Laboratory of Molecular Biology in Medical Sciences, Hangzhou, ZJ, China; b Department of Medical Oncology, The Second Affiliated Hospital, Zhejiang University School of Medicine, Hangzhou, ZJ, China; c Biological Systems and Engineering Division, Lawrence Berkeley National Laboratory, Berkeley, CA, USA; d Department of Hematology, Zhongnan Hospital, Wuhan University, Wuhan, China; e Berkeley Biomedical Data Science Center, Lawrence Berkeley National Laboratory, Berkeley, CA, USA; f Texas A&M Institute for Genome Sciences and Society, Texas A&M University, College Station, TX, USA; g Department of Molecular and Cellular Medicine and Department of Biochemistry & Biophysics, Texas A&M University, College Station, TX, USA; h Zhejiang Provincial Clinical Research Center for CANCER, Hangzhou, ZJ, China; i Cancer Center, Zhejiang University, Hangzhou, ZJ, China

**Keywords:** Colorectal tumor susceptibility, azoxymethane, genome-wide association study, gut microbiome, conditional knockout mouse, DUOX2

## Abstract

The insights into interactions between host genetics and gut microbiome (GM) in colorectal tumor susceptibility (CTS) remains lacking. We used Collaborative Cross mouse population model to identify genetic and microbial determinants of Azoxymethane-induced CTS. We identified 4417 CTS-associated single nucleotide polymorphisms (SNPs) containing 334 genes that were transcriptionally altered in human colorectal cancers (CRCs) and consistently clustered independent human CRC cohorts into two subgroups with different prognosis. We discovered a set of genera in early-life associated with CTS and defined a 16-genus signature that accurately predicted CTS, the majority of which were correlated with human CRCs. We identified 547 SNPs associated with abundances of these genera. Mediation analysis revealed GM as mediators partially exerting the effect of SNP UNC3869242 within *Duox2* on CTS. Intestine cell-specific depletion of *Duox2* altered GM composition and contribution of *Duox2* depletion to CTS was significantly influenced by GM. Our findings provide potential novel targets for personalized CRC prevention and treatment.

## Introduction

Colorectal cancer (CRC) is the third most common cancer and the second most common cause of cancer death in the United States.^1^ It is well-documented that the risk of developing CRC is influenced by both genetic and environmental factors. Approximately 25% of the CRC patients have a genetic predisposition with 5% of CRC being inherited;^2–5^ about 70% CRCs are sporadic, which may be linked to an interaction between genetics and environmental factors such as dietary habits, cigarette smoking, and alcohol consumption,^6–8^ Genome-wide association studies (GWAS) in both humans and mice suggest that nonfamilial CRC susceptibility results from the interaction among multiple small-effect alleles.^2,3,5,9,10^ About 100 genetic susceptibility regions related to CRC risk have been identified with GWAS.^2,3^

Strong evidence indicates that the abundance and composition of gut microbiome (GM) vary among individuals and determine the development of many diseases including cancer,^11–13^ Metagenome-wide association studies aim to identify the host microbes that are associated with diseases.^14^ An increasing number of studies suggest that the GM is directly involved in colorectal carcinogenesis,^15–17^ *Enterococcus faecalis*, *Bacteroides fragilis*, *Escherichia coli*, and *Fusobacterium nucleatum* were found to be enriched in the feces of CRC patients and to promote the occurrence of CRC.^17,18^ However, understanding the impact of host genetics, GM, and their interactions on the variability in CRC susceptibility remains limited.^19,20^ Direct human studies face a huge challenge due to the complex interplay among host genetics and GM in the context of uncontrollable environmental exposures and lifestyles.

Mouse models have contributed greatly to our understanding of cancer biology.^21^ The azoxymethane (AOM)-induced CRC mouse model has been widely used since AOM induces tumors in the distal mouse colon that resemble sporadic CRC of the descending colon in humans both histologically and molecularly.^22,23^ Moreover, researchers have identified many genetic loci that are susceptible to AOM-induced CRC by crossing resistant and susceptible mice.^9,10^ However, genetic variants distinguishing these commonly used strains are limited, and additional variants that are relevant to human CRC are likely missing. The Collaborative Cross (CC) mouse resource was established by combining the genomes of eight genetically diverse founder strains, which contains a level of genetic diversity on par with the human population,^24–26^ The genetic variants are randomly distributed across the genome and are roughly twice the number of common single nucleotide polymorphisms (SNPs) as present in the human population.^24^ Many studies have also reported phenotypic diversity including spontaneous tumor development across CC mice.^27–35^

In this study, using CC mice together with an AOM-induced CRC protocol, we aimed to identify genetic and microbial determinants of AOM-induced colorectal tumor susceptibility (CTS) and investigate host gene-microbiota interactions contributing to colorectal tumorigenesis and their implications in human CRC using publicly available datasets including The Cancer Genome Atlas (TCGA) dataset.

## Results

### Variation in AOM-induced CTS across 30 CC strains

**Figure 1.**
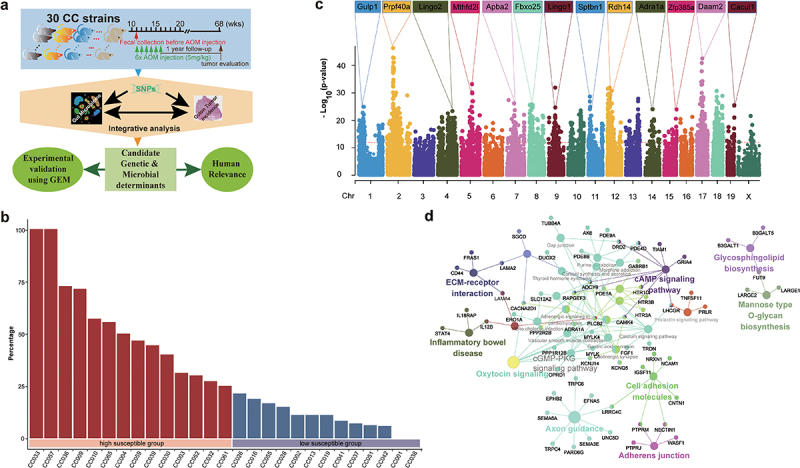
Identification of genetic variations and candidate genes associated with colorectal tumor susceptibility in CC mice.

To investigate AOM-induced CTS, 426 mice across 30 CC strains were intraperitoneally injected with 5 mg/kg AOM weekly for six weeks (Figure 1(a)). All mice were monitored up to 1 year after the last injection. Finally, 355 mice were sacrificed to assess colon tumor development. CC008, CC021, CC038 strains were excluded from study since the number of mice in each strain was less than 6. Colorectal tumors were observed in 25 out of 27 CC strains (Supplemental Table S1). We didn’t observe significant differences in tumor incidence between male and female mice as previous studies on common laboratory strains.^26^ Overall, the size of majority of tumors was small (about 1 mm of diameter), and number of tumors per mouse was few (<5). But we found that the incidence of AOM-induced colorectal tumorigeneses ranged from 100% of mice from CC033 and CC057 strains to none of mice from CC001 and CC040 strains, suggesting that host genetics control AOM-induced colorectal tumors. Therefore, the tumor incidence was the only phenotype to be focused on. 14 strains whose tumor incidence was higher than 25% were defined as high CTS group, while the remaining 13 strains were defined as low CTS group (Figure 1(b)).

(a) Illustrative graphics for study design. (b) AOM-induced colorectal tumor incidence in 27 CC strains. CC strains were divided into high susceptibility and low susceptibility groups based on their incidence. (c) Manhattan plot for the genetic association analysis of AOM-induced colorectal tumor in CC mice. The – log_10_(p-value) is shown for SNPs ordered based on genomic position. Representative candidate genes located in each QTL are listed. (d) Network plot for Kyoto Encyclopedia of Genes and Genomes (KEGG) pathway analysis of candidate susceptible genes.

### Genetic factors associated with AOM-induced CTS and their implication in human CRC

**Figure 2.**
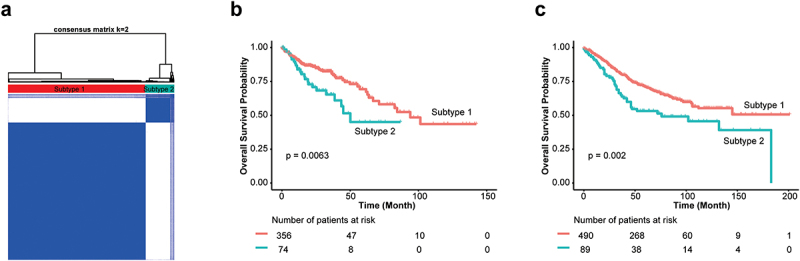
Subtyping human CRCs based on 334 CTS candidate genes.

We performed GWAS across 27 CC strains and identified 4417 of 94,909 SNPs significantly associated with CTS (*p* < 10^−12^) (Figure 1(c), Supplemental Table S2), containing 936 know human genes, 334 of which transcriptionally altered in human CRCs using TNMplot^36^ (Supplemental Figure S1, Supplemental Table S3). Additionally, we used a cross-validation (leaving one strain out) approach to confirm these identified SNPs and candidate genes (Supplemental Figure S2). Kyoto Encyclopedia of Genes and Genomes (KEGG) revealed that 936 genes were significantly enriched in cGMP-PKG and calcium signaling pathway among others (Figure 1(d), Supplemental Table S4). These pathways have been reported to play important roles in CRC development. To further assess clinical values of 334 candidate CTS genes, we clustered CRC patients based on their transcriptional expression in cancer and found that CRC patients were clustered into two groups that was determined by the optimal perceptual separation of consensus matrix in TCGA Colon Adenocarcinoma (TCGA-COAD) dataset (Figure 2(a), Supplemental Figure S3), which are significantly associated with overall survival (OS) (Figure 2(b)). Importantly, applying this pre-built classifier model to the validation cohort (GSE39582) subtyped patients into two groups that had significant differences in OS with the same directionality (Figure 2(c)). These findings suggest that the candidate genes identified in CC mice have clinical implications in human CRC.

(a) Consensus clustering model for CRC patient subtypes discovery and inference. (b, c) Subtype specific patients in TCGA-COAD (b) and GSE39582 (c) cohort show significant difference in overall survival.

### Gut microbiota community structures correlated to CTS

**Figure 3.**
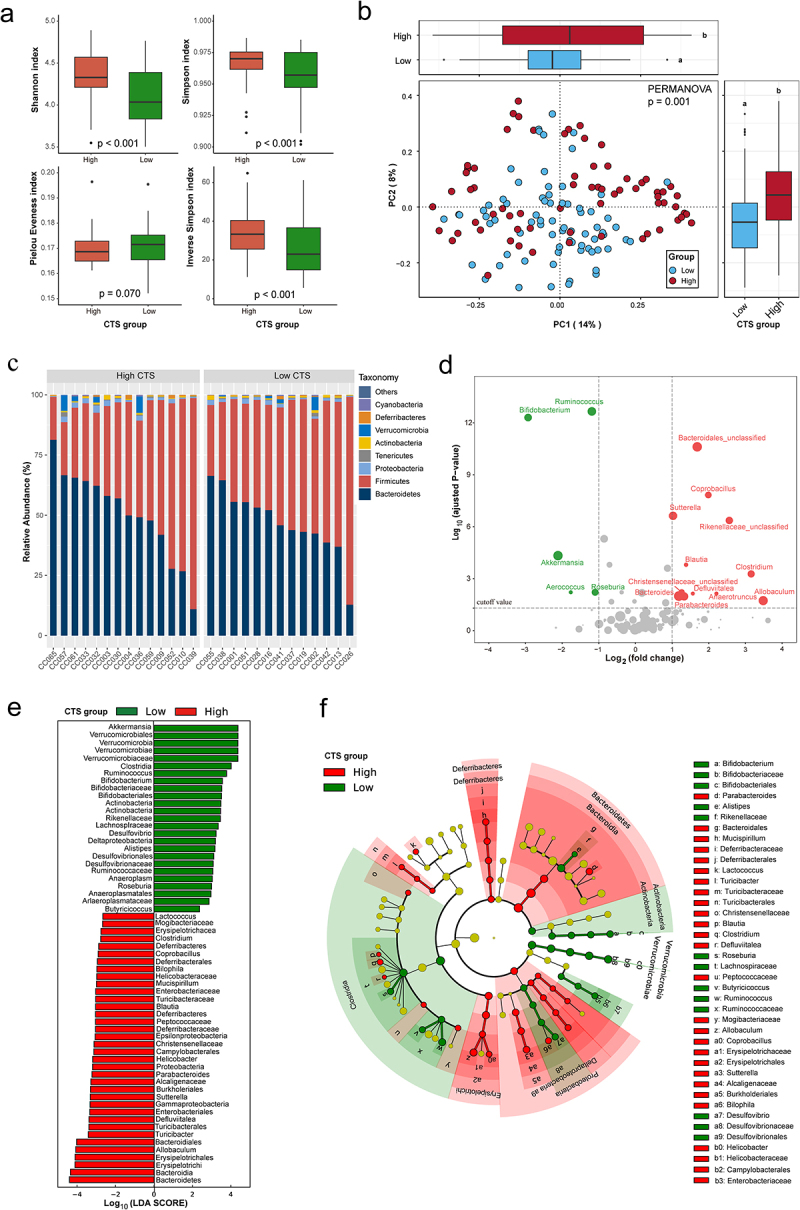
Differences in gut microbiome between high and low CTS group.

Strong evidence showed that the GM plays a vital role in CRC development.^17,18,37^ Therefore, we next determined whether any specific members of the early life GM could serve as biomarkers for predicting AOM-induced CTS. The reads from 16S rRNA sequencing analysis of fecal samples collected before AOM treatment from all 27 CC strains were mapped to 4364 OTUs corresponding to 157 bacterial genera (Supplemental Table S5). Compared to low CTS group, high CTS group had significantly higher richness and diversity of microbiota assessed by Shannon, Simpson and Inverse Simpson indices (*p* < .001, Figure 3(a)). Principle coordinates analysis (PCoA) based on Bray–Curtis dissimilarity index (beta diversity) revealed a significant difference in GM composition and abundance between low and high CTS groups (PERMANOVA: *p* = .001, Figure 3(b)).

(a) Alpha diversity boxplot between high and low CTS group. (b) PCoA using Bray–Curtis metric distances of beta diversity. The *p* value was obtained from PERMANOVA test of significant difference between low and high CTS groups based on Bray–Curtis dissimilarity. (c) Difference in abundance at order level between high and low CTS group. (d) Volcano plot showing genera enriched in high and low CTS group. Each dot represents a single genus. Genera significantly enriched in high and low CTS group are indicated by red and green dot, respectively (False discovery rate < 0.05 and | log_2_(FC) |>1.0). (e) Histogram representation of differentially abundant genus between high and low CTS group identified by Linear Discriminant Analysis (LDA) effect size (LEfSe). 26 genera were significantly enriched for their respective groups (Kruskal-Wallis test, *p* < 0.05, LDA score > 2). (f) Cladogram representation of 26 genera from Figure 3(e). The green highlighted the genera enriched in low CTS group; the red highlighted the genera enriched in high CTS group.

At the order level, we observed similar community structures between low and high CTS groups (Figure 3(c)). At the genus level, the abundances of 29 genera were significantly different between high and low CTS group (adjusted *p* < .05, Figure 3(d), Supplemental Table S6). To validate these findings, we conducted high dimensional class comparisons using linear discriminant analysis (LDA) effect size (LEfSe) that detected many more differences in the predominance of bacterial communities between high and low CTS groups. The LDA showed a clear alteration of the microbiota characterized by significantly higher *Allobaculum*, *Sutterella*, and *Turicibacter* levels and lower *Roseburia* level in high CTS group (LDA score > 2, Figure 3(e)). We consistently found that *Akkermansia*, *Ruminococcus*, *Desulfovibrio*, and *Bifidobacterium* genera were enriched in low CTS group, while *Blautia*, *Coprobacillus*, and *Parabacteroides* genera were enriched in high CTS group (Figure 3(d,e)). According to the bacterial community profiles, a hierarchical heatmap indicated that the most significantly different genera showed different patterns between high and low CTS groups (Figure 3(f)). Collectively, these findings suggest that early life gut microbiota community structures are significantly correlated with AOM-induced CTS in CC mice.

### Identification of a gut microbial signature for predicting CTS

**Figure 4.**
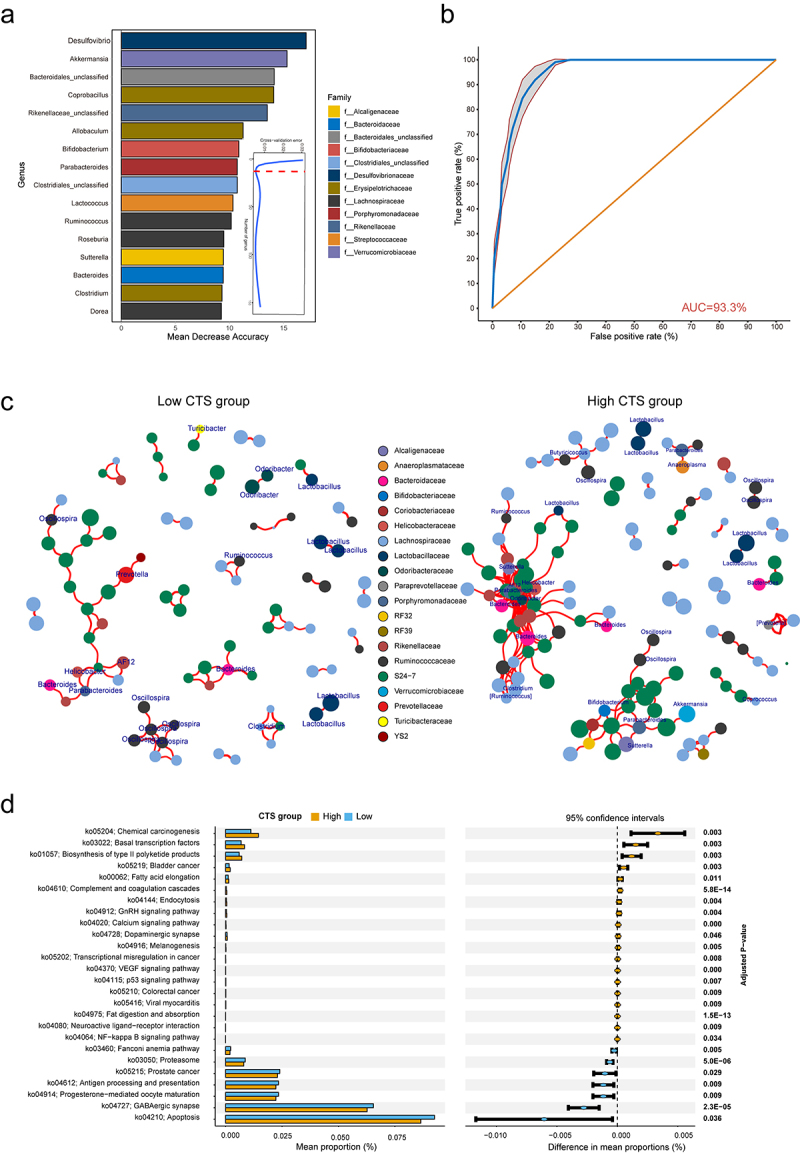
The core genera for predicting CTS and their related function analysis.

To explore the predictive value of the early life GM in CTS, we built a random forest classifier to distinguish the high and low CTS groups at the genus levels. We carried out 10-fold cross-validation with five repeats to evaluate the importance of genera. The optimal model was created utilizing 16 genera (*Desulfovibrio*, *Akkermansia*, *Bacteroides*, *Sutterella*, *Roseburia*, *Ruminococcus*, *Lactococcus*, *Clostridiales_unclassified*, *Parabacteroides*, *Bifidobacterium*, *Allobaculum*, *Rikenellaceae_unclassified*, *Coprobacillus*, *Bacteroidales_unclassified*, *Clostridium*, *Dorea*), which produced the best discriminatory power (Figure 4(a)). Random forest classification based on the relative abundance levels of 16 genera resulted in an area under the Receiver Operating Characteristic curve (AUC) of 0.933 (Figure 4(b)). These results suggest that specific gut microbes in early life may serve as biomarkers for predicting risk susceptibility to AOM-induced colon tumors.

(a) Random-forest analysis identified bacterial taxa that accurately predict high and low CTS. Biomarker taxa are ranked in descending order of importance to the accuracy of the model. The inset represented ten-fold cross-validation error as a function of the number of input genera used to differentiate high and low CTS. The dashed red line indicated the optimal cutoff for number of genera selected in the random forest predictive model. (b) ROC of the random forest model constructed using the 16 genera. The 95% confidence intervals were shown as shaded areas. (c) Microbial co-occurrence network was constructed based on samples of the low (*n* = 180, left panel) or high (*n* = 162, right panel) CTS group. The network connections were based on correlation test (*p* < 0.05 and Pearson’s correlation coefficient > 0.5). Genera nodes were colored according to families. (d) TaxFun2 analysis defines the difference of KEGG pathways between high and low CTS group.

To further understand the microbial contributions to CTS, we examined the microbiota network and bacterial function prediction. We constructed a cross-niche microbial network for each CTS group and found large differences in the microbial networks (Figure 4(c)). In low CTS group, the interactions among genera occurred mostly within same family with small cluster size, whereas in high CTS group, the gut microbiota formed two densely connected modules composed of diverse genera (Figure 4(c)). One module was centered on “beneficial microbiota”, including *Ruminococcus, Akkermansia*, and *Bifidobacterium*. The other module was centered on *Bacteroides, Sutterella*, and *Parabacteroides*. This result suggests that there are less microbial connections in low CTS mice than in high CTS mice, indicating that host genetics associated with CTS impact microbial networks.

We further performed Tax4Fun2 analysis to predict and test the difference in metagenome functions of KEGG pathways between high and low CTS groups. The low CTS group was enriched with pathways associated with antigen processing and presentation, progesterone-mediated oocyte maturation, GABAergic synapse, and apoptosis. In contrast, the pathways related to chemical carcinogenesis, transcriptional misregulation in cancer, p53 signaling pathway, colorectal cancer, and NF-kappa B signaling pathway increased in high CTS group (adjusted *p* < 0.05, Figure 4(d)). Taking all findings together, we conclude that host GM plays a critical role in the susceptibility to AOM-induced colorectal tumor in CC mice.

### GM as a mediator between genetic variants and CTS

**Figure 5.**
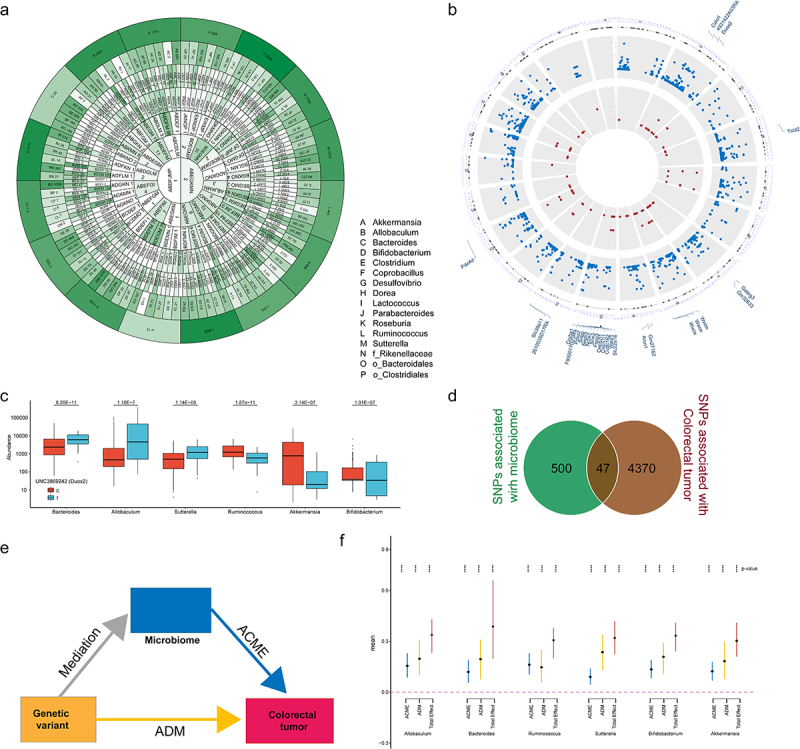
Gut microbiome partially mediates the effects of genetics on CTS.

To investigate whether genetic variations control CTS through GM, we first identified the genetic variants associated with the 16 genera that predicted CTS (Supplemental Table S7). GWAS analysis discovered 547 SNPs significantly associated with the abundance of 5 or more of 16 genera (*p* < 10^−6^, Figure 5(a–c), Supplemental Table S8). Among them, 47 SNPs overlapped with CTS SNPs (Figure 5(d)). 5 (*DUOX2*, *PDE4D*, *WASF1*, *SOBP* and *SLC39A11*) of corresponding genes for these 47 SNPs were among 334 CRC transcriptionally altered genes. To further investigate the potential causal links between GM and CTS, we then performed mediation analysis (Figure 5(e)) to determine whether genetic variants indirectly contribute to CTS by controlling the abundance of the 16 genera. Mediation analysis revealed that *Ruminococcus*, *Akkermansia*, *Allobaculum*, *Bacteroides*, *Sutterella*, and *Bifidobacterium* function as mediators for the effect of SNP UNC3869242 within *Duox2* on CTS (Figure 5(f)). Similar findings were observed for other 4 gene SNPs (Supplemental Figure S4). These results indicate that the effect of genetic variants on CTS is at least partially mediated by the GM, suggesting that the host genetics and GM interact to play an important role in CTS.

(a) The Venn diagram showed the microbiome-GWAS profile of 16 selected genera. Each compartment indicated the included selected genera and the number of microbiome-related SNPs in it. A SNP is considered related to microbiome when *p* < 10^−6^. All number values were included. (b) Circular visualization of chromosomal positions for all SNPs related to selected only genetic variants (SNPs) significantly associated with both the abundance of the five and above of 16 genera and colorectal tumorigenesis susceptibility. The outer layer shows chromosome location and candidate genes within each genetic locus are listed on the outside of the outer ring. The second layer (blue) shows SNPs related to colorectal tumorigenesis susceptibility (Mann-Whitney U test, *p* < 10^−6^). The inner layer (red) shows the microbiome related SNPs for ≥ 5 genera. (c) Boxplot showing *Duox2* (UNC3869242)-specific association with *Bacteroides*, *Allobaculum*, *Sutterella*, *Ruminococcus*, *Akkermansia*, and *Bifidobacterium* abundance according to its genotype. (d) Venn diagram showing 47 SNPs related to selected only genetic variants (SNPs) significantly associated with both the abundance of the five and above of 16 genera and colorectal tumorigenesis susceptibility. (e) Microbial genera mediate the effect of host genetics on colorectal tumorigenesis susceptibility. (f) Six microbial genera were identified as mediators between genetic variants and colorectal tumorigenesis susceptibility. The estimate score and 95% CI for microbial genera associated with colorectal tumorigenesis susceptibility was calculated by mediation analysis.

### GM composition alteration in Duox2 deficient mice

*Duox2* is one of five candidate genes that may control both CTS and the abundance of many genera and are transcriptionally altered in human CRCs. Therefore, we next investigated the role of *Duox2* in CRC development and GM composition.

**Figure 6.**
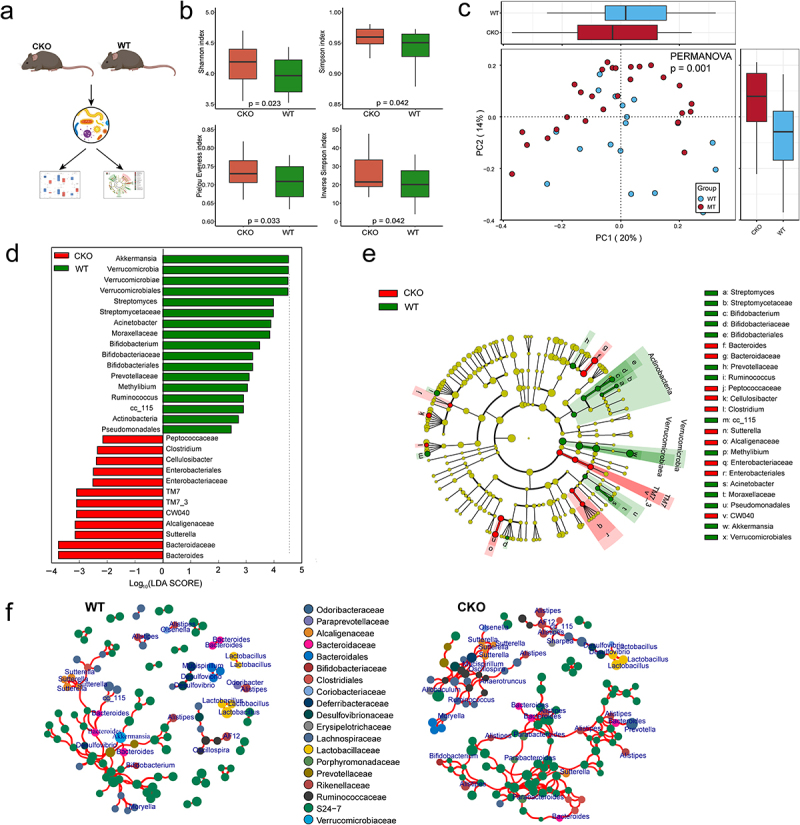
Depletion of *Duox2* alters gut microbiome structure in mice.

DUOX2 is a critical modulator in mutualistic host-microbiota interactions that are fundamental in maintaining gut immune homeostasis, which has been reported to be involved in colitis-associated colorectal tumorigenesis.^38,39^ Our findings in CC mice suggest contribution of *Duox2* to colorectal tumor development is possible via modulation of the GM composition. Experimental models of colitis-associated tumorigenesis are indispensable to improve our understanding of intestinal pathophysiology and host-microbiota interactions. Inflammation-dependent tumor growth can be investigated by combining the administration of AOM with the inflammatory agent dextran sodium sulfate (DSS) in drinking water. AOM/DSS Chemically induced model that replicates the aspect of colitis associated tumorigenesis is widely used.^40,41^ The knockout of *Duox2* gene in mice causes both hypotension and bradycardia basally.^39^ To exclude the possibility that these deficiencies confound our findings, we generated intestine-specific *Duox2*-deficient mice by crossing *Duox2*^fl/fl^ mice with *Villin-Cre* transgenic mice, which specifically deleted *Duox2* from intestinal epithelia (denoted as *Duox2* CKO mice) to investigate the effect of *Duox2* deficiency on composition of GM (genotype by PCR shown in Supplemental Figure S5). 16S rRNA sequencing analysis of the feces from *Duox2* wildtype (WT) and CKO mice (Figure 6(a)) revealed that depletion of *Duox2* in intestinal cells significantly altered both alpha and beta diversity in the GM (Figure 6(b,c)). Additionally, we observed significant taxonomic differences in the feces microbiome between *Duox2* WT and CKO mice (Figure 6(d), Supplemental Table S9). The relative abundance of *Bifidobacterium* and *Akkermansia* genera were significantly reduced, while the abundance levels of *Bacteroides* and *Sutterella* genera were significantly increased in CKO mice compared to WT mice (Figure 6(d,e)). Moreover, we found a large difference in the microbial networks between *Duox2* WT and CKO mice. Contrary to weaker gut microbiota interactions in *Duox2* WT mice, the gut microbiota in *Duox2* CKO formed two densely connected modules composed of diverse genera (Figure 6(f)). One module was centered on “beneficial microbiota”, including *Lactobacillus* and *Bifidobacterium*. The other module was centered on *Bacteroides*, *Sutterella*, and *Parabacteroides*, which are reported to be related to CTS. Thus, we considered this module to consist of “pathogenic microbiota” (Figure 6(f)). Collectively, we conclude that *Duox2* may regulate the composition of GM.

(a) The microbiota structure of *Duox2* ^*fl/fl*^ (WT) and *Duox2*^*fl/fl*^ *: Vilin-cre* (CKO) mice. (b) Alpha diversity boxplot between WT and CKO group. (c) Principal component analysis and maximally collapsing metric learning of WT and CKO. (d, e) Differentially abundant taxa between low and high group analyzed by Linear Discriminant Analysis (LDA) effect size (LEfSe) were projected as histogram (d) and cladogram (e). (f) Microbial co-occurrence network constructed based on samples of WT and CKO group. The network connections are based on correlation test (*p* < .05 and Pearson’s correlation coefficient > 0.5). Edge connection between genera is shown in black lines. Genera nodes are colored according to families.

### Interplay between DUOX2 and GM contributes to CTS

**Figure 7.**
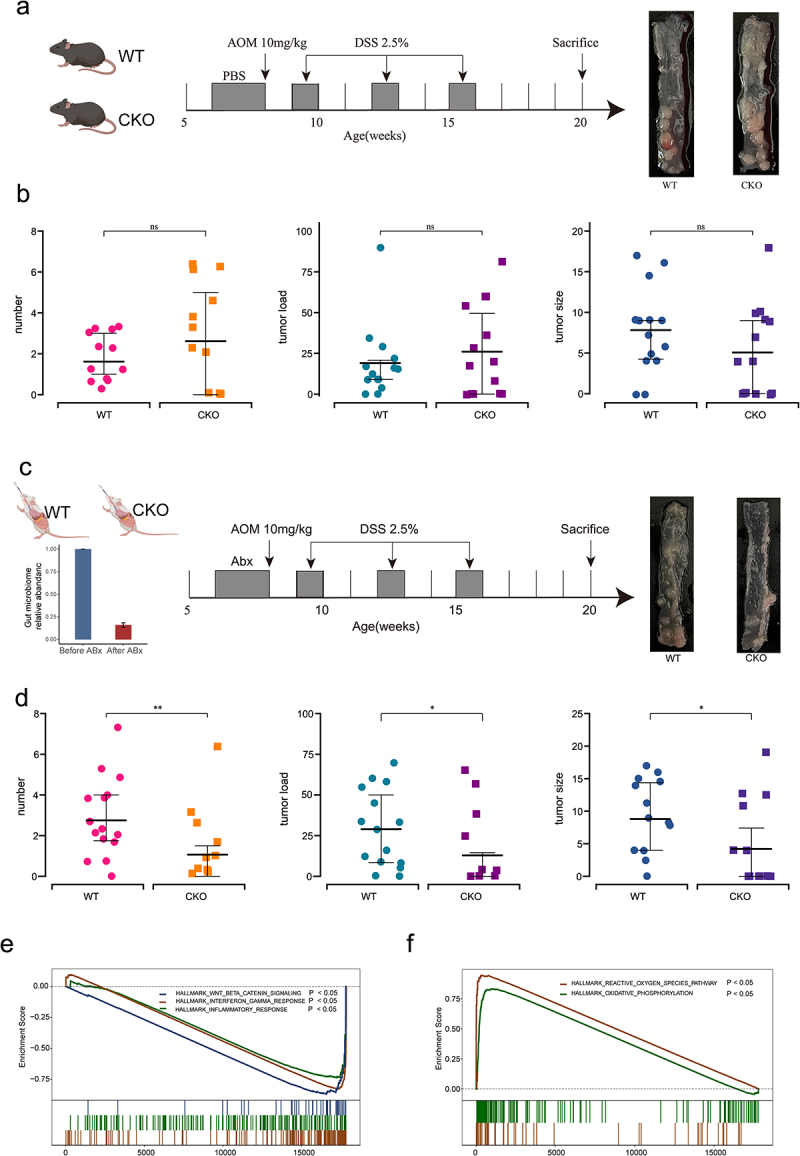
Interplay of *Duox2* with gut microbiota and contributed to colorectal tumorigenesis susceptibility.

We next investigated whether interplay between DUOX2 and GM contributed to CTS. *Duox2* WT and CKO mice were administered with AOM and DSS to establish a colitis-associated tumorigenesis (Figure 7(a)). There was similar CTS between CKO and WT mice evidenced by the number, size, and tumor load of macroscopic polyps between CKO and WT mice (*p* > .05, Figure 7(b), Supplemental Figure S6). Since significant alteration of the GM composition was found in *Duox2* deficient mice, we investigated the influence of GM on contribution of *Duox2* loss to CTS. Therefore, before treatment of AOM and DSS, mice were treated with antibiotic (ABX), 2 weeks prior to induction with AOM and DSS. The efficacy of ABX was verified (Figure 7(c)). After AOM and DSS administration, there was less CTS in *Duox2* CKO mice than WT littermates evidenced by significant lower number, size, and tumor load of macroscopic polyps (*p* < .05, Figure 7(d), Supplemental Figure S6). These data demonstrated that the contribution of DUOX2 depletion to colon tumorigenesis is influenced by GM.

(a) *Duox2*^*fl/fl*^ (WT) and Duox2^fl/fl^ Villin-Cre (CKO) mice were challenged with AOM and DSS to establish colitis-associated tumorigenesis, which mimics the colitis-related tumorigenesis. (b) After AOM and DSS administration, there is similar colorectal tumorigenesis in CKO mice (*n* = 14) and WT littermates (*n* = 14) evidenced by similar the number, size and tumor load of macroscopic polyps between CKO mice and WT group. (c) Mice were treated with antibiotics (ABX) two weeks prior to induction with AOM and DSS. The efficacy of ABX was verified. (d) After AOM and DSS administration, less colorectal tumorigenesis susceptibility was seen in CKO mice (*n* = 15) than WT littermates (*n* = 16) via comparing the number, size, and tumor load of macroscopic polyps. (e) GSEA plot showed that conditional knockout Duox2 would lead to downregulate Wnt/β-Catenin pathway, inflammatory response and interferon-γ response pathway. (f) GSEA plot showed that conditional knockout Duox2 would lead to upregulate reactive oxygen species pathway and oxidative phosphorylation pathway.

To discover underlying mechanisms by which *Duox2* contributes to colorectal tumor development, we conducted RNA-Seq analysis of colon-derived RNA from *Duox2* WT and CKO mice. Depletion of *Duox2* led to the differentiated expression of 48 genes (adjusted *p* values < .05 and |log_2_(FC)| > 1.0; Supplemental Figure S7, Supplemental Table S10). Gene set enrichment analysis (GSEA) revealed that the knockout of *Duox2* resulted in downregulation of the Wnt/β-Catenin, inflammatory response and interferon-g response pathways and increased activation in reactive oxygen species and oxidative phosphorylation pathways (Figure 7(e,f)), all of which play an important role in both tumor development and gut microbiome composition.

### Evaluation of DUOX2 and its associated gut microbiota in human CRC

**Figure 8.**
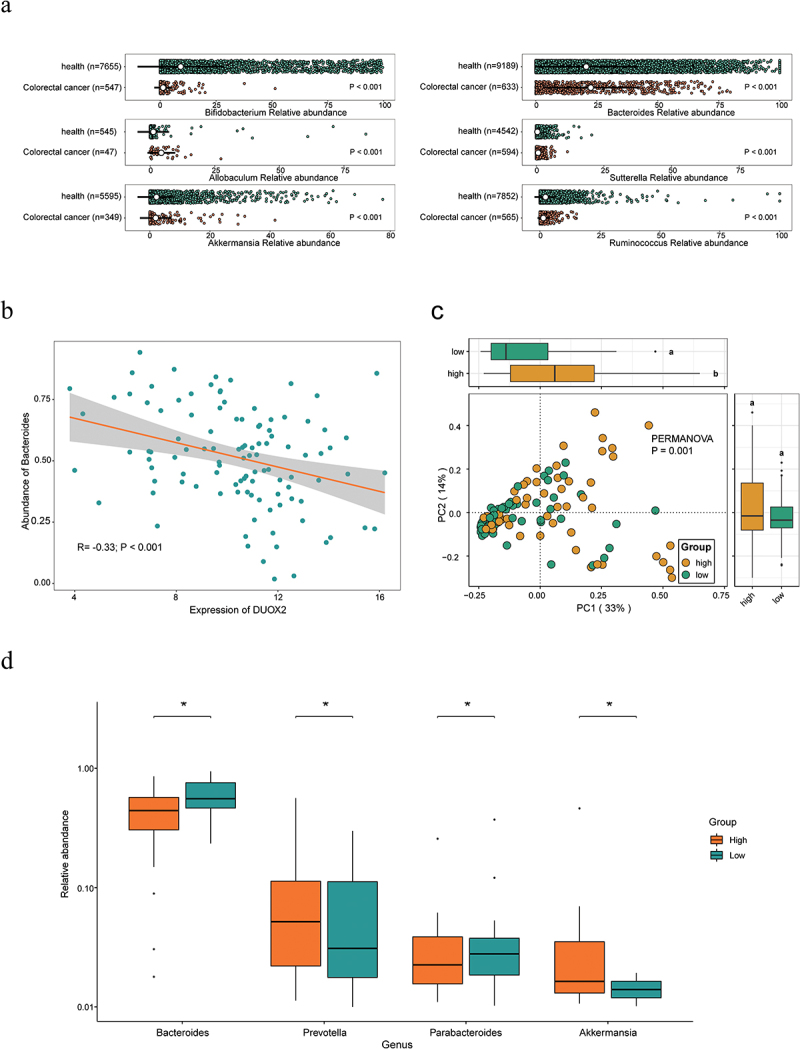
Validation of core different abundant taxa and the function of DUOX2 according to public data.

To evaluate DUOX2 and its associated gut microbiota in human CRC, we conducted a series of bioinformatics analysis. In the GMrepo database, we found that compared with healthy donors, patients with CRC had significantly lower levels of *Bifidobacterium*, *Ruminococcus*, and *Akkermansia* (*p* < 0.001), and higher levels of *Allobaculum*, *Bacteroides*, and *Sutterella* (*p* < .001, Figure 8(a)), consistent with our findings in CC mice.

(a) Compared with healthy donors, patients with colorectal cancer (CRC) had significantly lower levels of *Bifidobacterium* (*p* < .001), *Ruminococcus* (*p* < .001) and *Akkermansia* (*p* < .001), while the abundance of *Allobaculum* (*p* < 0.001), *Bacteroides* (*p* < .001), and *Sutterella* (*p* < .001) bacterial abundance significantly increased. (b) DUOX2 was significant negatively with Bacteroides abundance based on The Cancer Microbiome Atlas (TCMA) database. (c) 106 cases of colorectal cancer patients were divided into two groups with high expression (High) and low expression (Low) with the median of DUOX2 expression in colorectal cancer tissues. It was found that there was a significant difference in gut microbiome community between the two groups. Bray–Curtis distance for PCoA analysis and PERMAOVA for difference testing. The PCol interpretation rate was 33%, the PCo2 interpretation rate was 14%, and the PERMAOVA test (*p* = 0.001). (d) Boxplot showed that the abundance of *Akkermansia* and *Prevotella* was significantly higher in the high group than that in low group, while the abundance of *Bacteroides* and *Parabacteroides* were significantly lower in high group than that in low group.

In The Cancer Microbiome Atlas (TCMA),^42^ we found that *DUOX2* expression is significantly negatively correlated with *Bacteroides* abundance (Figure 8(b), Supplemental Table S11). To explore the association of *DUOX2* expression with the GM community, 106 cases of CRC patients were divided into two groups based on the median of *DUOX2* expression in CRC tissues, defined as *DUOX2* high and low expression groups (Supplemental Table S11). PCoA analysis revealed a significant difference in GM community between the two groups (*p* = .001, PERMANOVA, Figure 8(c)). The boxplot showed that the abundance of *Akkermansia* and *Prevotella* genera was significantly higher, while the abundance of *Bacteroides* and *Parabacteroides* genera was significantly lower in high group than that in low group (*p* < .05, Figure 8(d), Supplemental Table S11), consistent with our findings in *Duox2* CKO mice.

## Discussion

Using the CC mouse population-based model together with the AOM-induced colorectal tumor model, we deciphered the contribution of host genetics, microbiome, and their interactions to colorectal cancer risk. Many genetic and microbial factors were identified to control AOM-induced CTS. We provide evidence for interactions between host genetics and GM, and meditation effects of microbes on the genetic contribution to CTS in both population-based and gene knockout mouse models. We further demonstrated the genes and microbes identified in this CC mouse population study have an impact on human CRC.

This study showed that the CC mouse population, equivalent to a genetically diverse human population, provides a useful resource for revealing a genetic architecture of AOM-induce CTS. 4,417 SNPs corresponding to 936 known human genes, have been identified to be significantly associated with AOM-induced CTS, including the majority of the loci identified in previous studies.^9,10^ Especially our findings are comparable to a recent study in 40 mouse inbred strains,^10^ consistent with the report that CC mice captures 90% of genetic variations in laboratory inbred mice.^24^ The results from the CC mice extend the catalog of CTS genes that can be used to evaluate human CRC susceptibility. Additionally, 75 of 936 genes have been found to be associated with human CRCs by GWAS (Supplemental Table S3). Moreover, 334 of 936 genes have transcriptionally altered in human CRCs. Genetic variants in *Duox2* were found to be associated with human CRC through GWAS. Moreover, the transcriptional expression of human 334 genes classified CRC patients into two different prognostic groups. These suggest that the findings in this CC mouse population study can be translated into humans.

Overwhelming evidence suggests that the GM plays a critical role in CRC development.^16,18,43^ In this study, we found that the abundance levels of 16 genera were significantly associated with AOM-induced CTS. The relative abundance levels of 16 genera predict high or low CTS with AUC of 0.933. 13 of 16 genera have been reported to be altered in human CRC.^37,44^ Specifically, abundance of *Bacteroides* was elevated in colorectal cancer patients,^37^ consistent with our finding that *Bacteroides* was more abundant in high CTS group. Increased tumorigenesis has been reported in mice colonized with *Bacteroides* through an IL-23-dependent and STAT3-dependent manner associated with TH17 activation.^45^ *Bacteroides* are not all the same – a specific strain of *B. fragilis* produces a toxin that induces IL17. Also, we cannot exclude the possibility that there are other non-toxigenic strains that may promote tumorigenesis via other mechanisms. *Bifidobacterium* is more abundant in the low CTS group, consistent with previous reports that *Bifidobacterium* species have anti-cancer effects in CRC.^46^ Interestingly, *Bifidobacterium* is one of the most used probiotics and has also been reported to enhance the efficacy of cancer immunotherapies.^47^ Further characterization of these putative probiotic species identified in this study can offer promising potentials for preventative and therapeutic treatment of human CRC.

DUOX2 is a critical modulator in mutualistic host-microbiota interactions, which has been reported to be involved in colitis-associated colorectal tumorigenesis. Many reported studies have showed that the gut microbiota engages different signaling pathways including MyD88, p38 MAPK pathways to induce Dual Oxidase 2 (*DUOX2*) expression in the ileum and colon epithelium.^48^ Epithelium is a first line of defense against microorganisms in the gut. Reactive oxygen species (ROS) play an important role in controlling the normal gut microbiota and pathogenic bacteria. DUOX2 is an important source of hydrogen peroxide in the small and large intestine, and the gut microbiota can induce *DUOX2* expression.^48^ The microbiota activates TLR4 which in turn stimulates epithelial ROS through DUOX2. Increased epithelial ROS production is associated with pro-tumorigenic microbiota. Both an altered microbiota and epithelial ROS are essential for colonic tumorigenesis.^39^ In this study, we discovered dual functions of DUOX2 on CRC development, which provides a new insight into mechanisms of DUOX2 for tumor development. Depletion of DUOX2 significantly altered intestinal homeostasis through a decrease in “beneficial” gut microbes and an increase in “harmful” gut microbes. Moreover, we showed that GM influences the contribution of DUOX2 depletion to colon tumorigenesis in an inflammatory model of colon cancer. The interrelationship between DUOX2 and tumor-promoting microbiota requires a two-pronged strategy to reduce the risk of dysplasia in colitis-associated colorectal tumorigenesis.^49^ Although the causal effects of other genetic variants on GM and CTS still require further investigation, our findings suggest that host genetics and GM interactions play an important role in CRC development.

While this study successfully identified novel targets for CRC prevention and treatment, there were some limitations. First, we used 16S rRNA gene sequencing to study the metagenomic composition. A key limitation of 16S rRNA gene sequencing is the difficulty in determining the exact microbial species. Therefore, we limited our analysis to genera level analysis. One specific limitation of amplification-based 16S rRNA sequencing is the introduction of PCR and/or sequencing errors, that may result in sequence classification errors, and ultimately the detection of similar, but incorrectly identified microorganisms, or the mistaken belief that new microorganisms have been found. To address this problem, we used OTU clustering for this study since we explored the large-scale biodiversity across CC strains to link the microbiome to colon tumor susceptibility. Future studies should incorporate metagenomic sequencing to detect less abundant genera that may play an important role in CTS. Second, our study focused on the role of *Duox2* in mediating the effects of GM on CTS. In this study, we identified additional candidate genes that are likely to contribute to CTS which should be further investigated.

In conclusion, we used the population-based CC mouse model together with AOM-induced CRC protocol to identify many existing and new genetic and microbial determinants of AOM-induced CTS and used a conditional mouse knockout model to validate the importance of host gene-microbiota in colorectal tumorigenesis. DUOX2 plays dual functional roles in CRC development, so clinically targeting DUOX2 for the treatment of gut associated diseases must consider both the autonomous and non-autonomous functions. Overall, the findings in this study shed light on directions for future applications in personalized CRC prevention and treatment.

## Materials and Methods

### CC mice and AOM treatment

All CC mice were purchased from the Systems Genetics Core Facility at the University of North Carolina. Mice were acclimated at the Lawrence Berkeley National Laboratory (LBNL) for 8 weeks prior to the initiation of breeding. Offspring were weaned at 21 days of age. Mice were raised in groups of 2–5 mice per cage in individually ventilated cages in a light-controlled room with 12:12 h light/dark cycle. All mice were maintained on standard chow (PicoLab Rodent diet 5053). Since 10 mg/kg of AOM is lethal to some CC strains,^50^ 426 11-week-old mice were injected intraperitoneally once weekly with 5 mg/kg of AOM (Sigma, St. Louis, MO) for six weeks for tumor induction and were monitored for one year (Figure 1(a)). Finally, a total of 355 surviving mice from 30 CC strains were used in this study (Supplemental Table S1). Three strains with less than 6 mice were excluded from analysis. This study was carried out in strict accordance with the Guide to the Care and Use of Laboratory Animals of the National Institutes of Health. The Animal Welfare and Research Committee at Lawrence Berkeley National Laboratory approved the animal use protocol.

### GWAS on CTS

GWAS analysis has been described as previously.^30,50,51^ Briefly, CC strains were divided into high and low CTS groups based on tumor incidence. At each SNP, the Chi-Square test was used to assess the significance of associations between tumor susceptibility and allele types. *p* < 10^−12^ was criteria for significance based on permutation analysis. Putative candidate genes were defined as those genes containing a significant SNP within the boundaries of the gene sequence (http://www.informatics.jax.org/).

### 16S rRNA sequencing analysis of fecal samples

Fecal samples were collected from individual cages 16 h following cage change prior to the first AOM injection and were stored at −80°C until microbial analysis. At least four independent cages were sampled for each CC strain. We extracted genomic DNA from the homogenized fecal samples, amplified 16S rRNA by PCR using primers targeting the V4 region, performed 16S rRNA sequencing by Illumina MiSeq and analyzed the data as previously described.^30,50,51^ Briefly, the sequence reads were quality-filtered using QIIME2 (version 2019.7).^52^ Filtered reads were clustered into operational taxonomic units (OTUs), using an open reference picking process with a threshold of 97% similarity to the reference database (Greengenes OTUs (16S) v13_8). Mouse 16S rRNA sequence data from the CC mouse cohort, which was used in our previous study,^30^ is available on OSF (https://osf.io/jbt5g/).

### Association analysis of metataxonomics with CTS

The PERMANOVA method was used to test for differences in bacterial community composition, as quantified by weighted UniFrac distance between samples. Relative abundance was assessed using the most specific taxonomic assignment available for each OTU. Taxa were selected for testing if the mean abundance exceeded 1% among the samples to be analyzed. Differences in the alpha diversity and microbiome features between high and low CTS groups were assessed by Mann–Whitney test and/or linear discriminant analysis of effect size (LEfSe). The random forest classification model with 10 cross-validation iterations was used to evaluate the effectiveness of the microbial signature for predicting CTS. The microbial co-occurrence networks were established using Spearman’s rank correlations and visualized in ggplot2 package (version 3.2.1). Tax4Fun2 (version 1.1.5) was used to predict metagenome functional content and test the difference of Kyoto Encyclopedia of Genes and Genomes (KEGG) pathways between high and low CTS groups.

### Mediation analysis of GM between genetic variants and CTS

Mediation analysis is a statistical model to determine whether the relationship between two variables (e.g., genetic variant and CTS) is mediated through a third variable (e.g., GM), which was performed using mediation package (version 4.4.7) and visualized using ggplot2 package (version 3.2.1) in R (version 3.5.0).

### Conditional knockout mice and AOM/DSS-induced colorectal tumorigeneses

We generated intestine-specific *Duox2*-deficient mice by crossing *Duox2* floxed (*Duox2*^*fl/fl*^) mice with *Villin-Cre* transgenic mice, resulting in deletion of *Duox2* exon 4–6 in the intestine cell. The feces were collected from *Duox2*^*fl/fl*^, *Villin-Cre* (CKO) and *Duox2*^*fl/fl*^ (WT) mice for 16S rRNA analysis. The GM composition was compared between CKO and WT mice. To minimize the effect of recolonization of the GI tract on tumor development after antibiotic (ABX) clearance of the microbiome, we used the AOM/DSS protocol to accelerate colorectal tumor development. For AOM/DSS-induced colorectal tumorigeneses, CKO and WT mice at 8-weeks of age were given an initial intraperitoneal injection of 10 mg/kg AOM. After 1 week, mice were given 3% DSS (MP Biomedicals) in drinking water for 1 week, followed by normal drinking water for 2 weeks. This cycle was repeated 3 times. In an additional study, before AOM/DSS induction, mice were treated with an antibiotic cocktail containing 1.86 mg/kg ampicillin, 1.86 mg/kg neomycin sulfate, 1.2 mg/kg metronidazole, and 0.96 mg/kg vancomycin in 300 μL double distilled water to deplete the gut microbiota.^53^ All mice were euthanized at 20-weeks of age for assessing tumor development. This study was approved by the Institutional Animal Care and Bioethical Committee of Zhejiang University.

### RNA-seq, differentiated gene, and gene enrichment analysis

Colon samples from WT and CKO mice were dissected and stored at −80°C. Total RNA was extracted using the mirVana miRNA Isolation Kit (Ambion) following the manufacturer’s protocol. RNA integrity was evaluated using the Agilent 2100 Bioanalyzer (Agilent Technologies). The libraries were constructed using TruSeq Stranded mRNA LTSample Prep Kit (Illumina) according to the manufacturer’s instructions. Then these libraries were sequenced on the Illumina sequencing platform (HiSeqTM 2500) and 125bp/150bp paired-end reads were generated. Raw reads were processed using Trimmomatic. Then, the clean reads were mapped to reference genome using hisat2. DESeq2 package (version 1.22.2) was used to identify differentially expressed genes (DEGs) between WT and CKO groups. The adjusted *p* values < .05 and |log_2_(fold change (FC))| > 1.0 were considered as significance. GSEA was used to analyze the functions of DEGs using ClusterProfiler package (version 3.8.1). The annotated gene collections downloaded from the Molecular Signatures Database (MSigDB v7.0) for H (hallmark gene sets).

### Data analysis of human colorectal cancer patients using public database

The difference in gene expression between normal and colon adenocarcinoma tissues was assessed using TNMplot (https://tnmplot.com/analysis/).^36^ TCGA-COAD transcriptome and clinical data were downloaded from the cBioPortal (https://www.cbioportal.org/).^54,55^ The GSE39582 cohort was downloaded from Gene Expression Omnibus (GEO) database. There was no additional modification in the downloaded data during our analyses. Human gut metagenomic data was obtained from GMrepo (https://gmrepo.humangut.info/home). GMrepo organizes the collected human gut metagenomes according to their associated phenotypes and includes all possible related data. Using this database, we identified differential genus abundance distribution between two phenotypes (health versus colorectal cancer) on a per-project basis for selected projects. TCGA intestinal microbiome data was obtained from The Cancer Microbiome Atlas (TCMA) (https://tcma.pratt.duke.edu/). TCMA was a statistical model to analyze the prevalence of microbial compositions for TCGA sequencing data across sample types. TCMA enables paired microbial-host transcriptome analyses that identify associations between microbial and host gene expression patterns and pathways. The level-3 RNA-sequencing data in the TCGA Colon Adenocarcinoma (TCGA-COAD) project were retrieved from UCSC xena (https://xenabrowser.net/).

### Statistical analysis

CRC patients were stratified using consensus clustering (ConsensusClusterPlus package in R, version 1.50.0) with k-means clustering, Pearson’s correlation and 500 bootstrapping iterations; and the optimal number of subtypes was determined by the consistency of cluster assignment (i.e., the consensus matrix). The difference in overall survival (OS) was assessed by Kaplan-Meier analysis (survminer package in R, version 0.4.8) and log-rank test (survival package in R, version 3.2–3). The t-test or Mann-Whitney test were employed to analyze differences in the abundance between two groups for normally or not normally distributed data, respectively. The *p* values were adjusted using the method of Benjamini and Hochberg with the function p.adjust in R. The correlation matrix between the gut microbiota and gene expression was generated using Pearson’s correlation coefficient. The p or adjusted *p* value < .05 was taken as statistically significant. All data analyses were performed, and plots were generated using R software (version 3.5.0).

## Supplementary Material

AOM_ColonTumor_GM_SupplementaryFigsTables.docx

## Data Availability

Mouse gut microbiome 16S rRNA gene sequencing data and RNA-Seq data from *Doux2* knockout mice are are available in the National Center for Biotechnology Information (NCBI) BioProject Repository (https://www.ncbi.nlm.nih.gov/bioproject) under the BioProject “PRJNA802804”. Mouse gut microbiome 16S rRNA gene sequencing data from CC mouse cohort is available on OSF (https://osf.io/jbt5g/).^30^ The mouse data has been included in Supplemental Table S1. The numeric counts table at genus level and corresponding taxonomic classifications have all been included as Supplemental Table S5.
