# Supplementary material for: Genetic and microbial determinants of azoxymethane-induced colorectal tumor susceptibility in Collaborative Cross mice and their implication in human cancer
Source: Gut Microbes. 2024 Apr 24;16(1):2341647. doi: 10.1080/19490976.2024.2341647 (PMC11057575; doi:10.1080/19490976.2024.2341647)
Supplement: AOM_ColonTumor_GM_SupplementaryFigsTables.docx [file KGMI_A_2341647_SM2212.docx]

Supplemental Figures and Tables

# Supplemental Figures


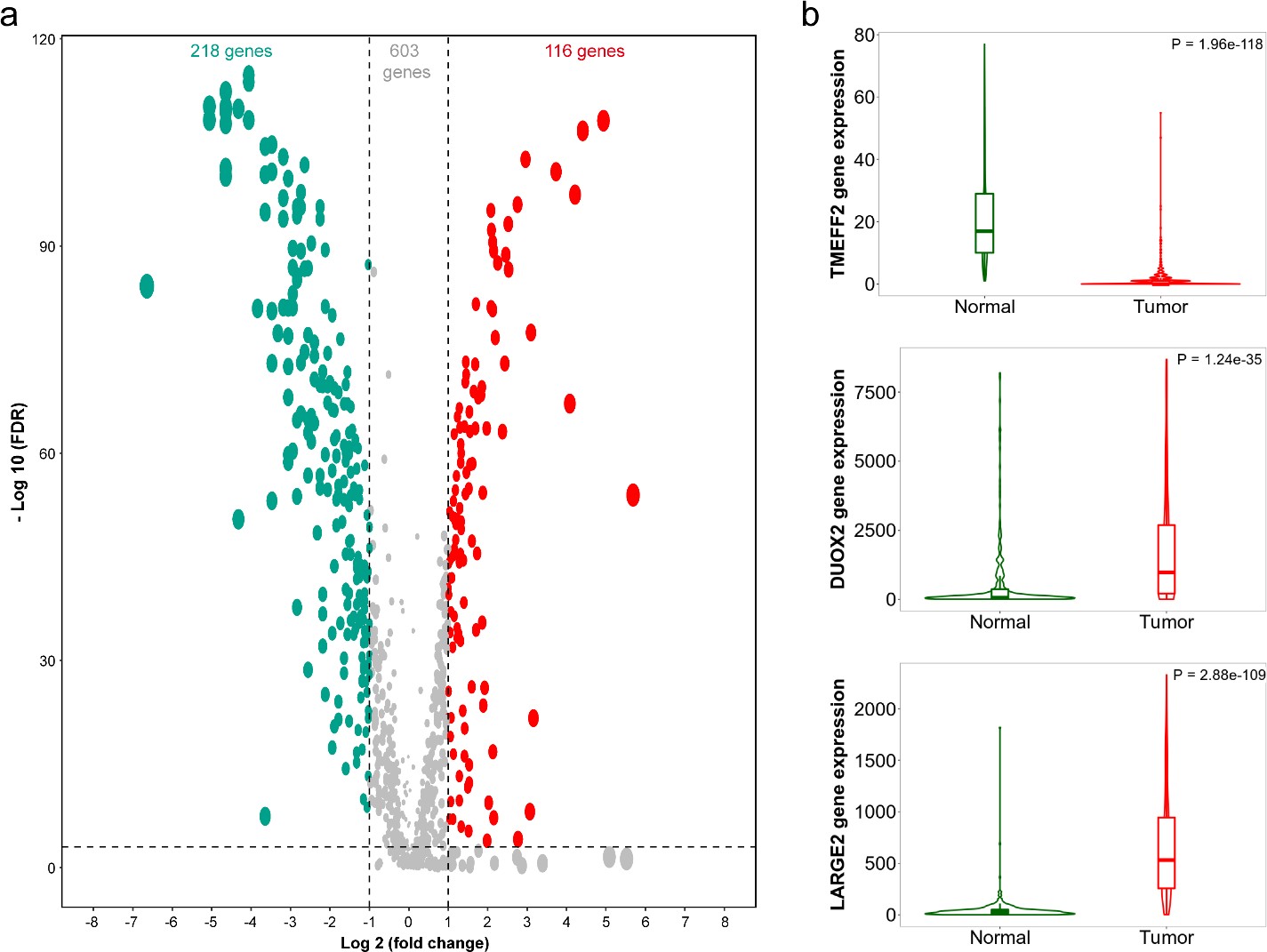


**Supplemental Figure S1** 334 of 936 CTS candidate genes are transcriptionally altered in human colon adenocarcinomas. (a) Volcano plot depicting the differentially expressed genes between adenocarcinomas and normal colon tissues (|log2FC| ≥1 and FDR ≤ 0.001). (b) Gene expression of representative genes in normal and tumor tissues.


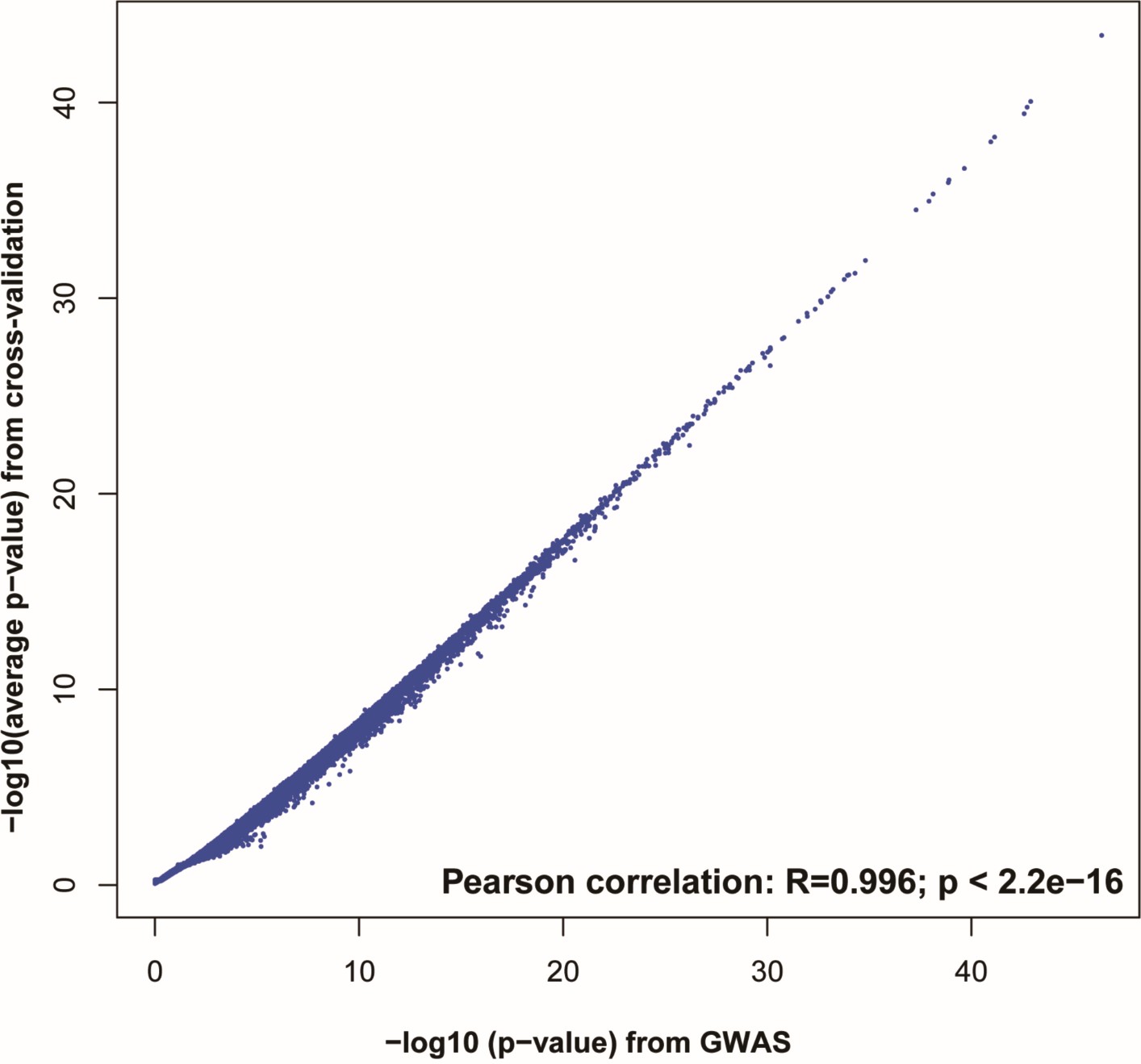


**Supplemental Figure S2** Correlations between the p-values obtained from GWAS analysis and cross-validation analysis confirmed the findings from GWAS.


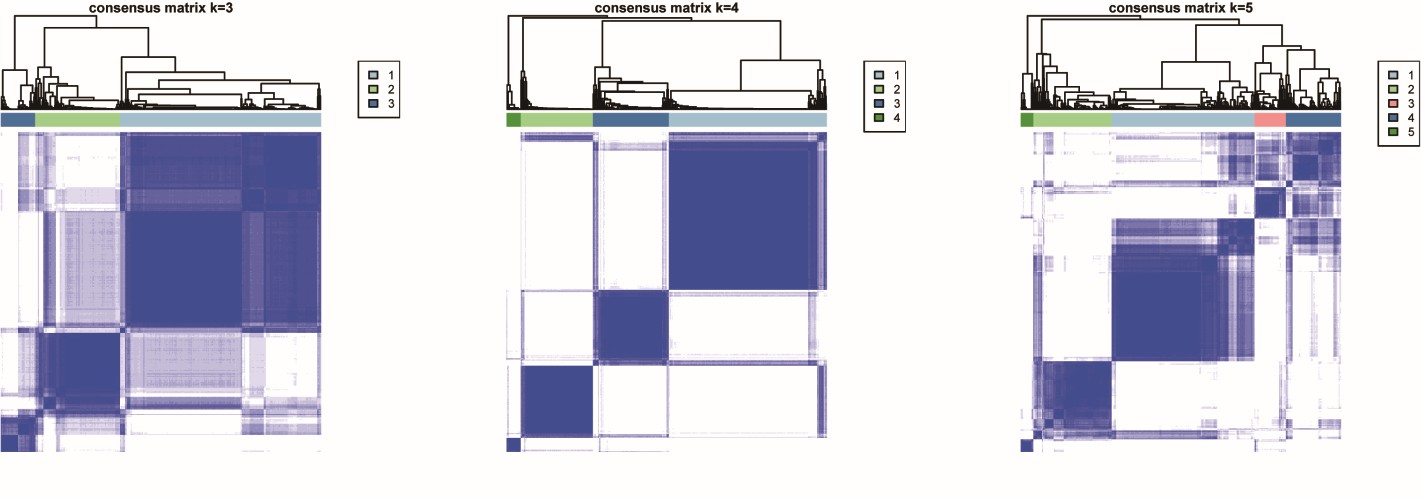


**Supplemental Figure S3** Classification of patients with colon cancer into different groups based on expression of 334 CTS candidate genes in TCGA-COAD cohort. Consensus classification heatmaps based on three (left panel) and four (right) clusters.


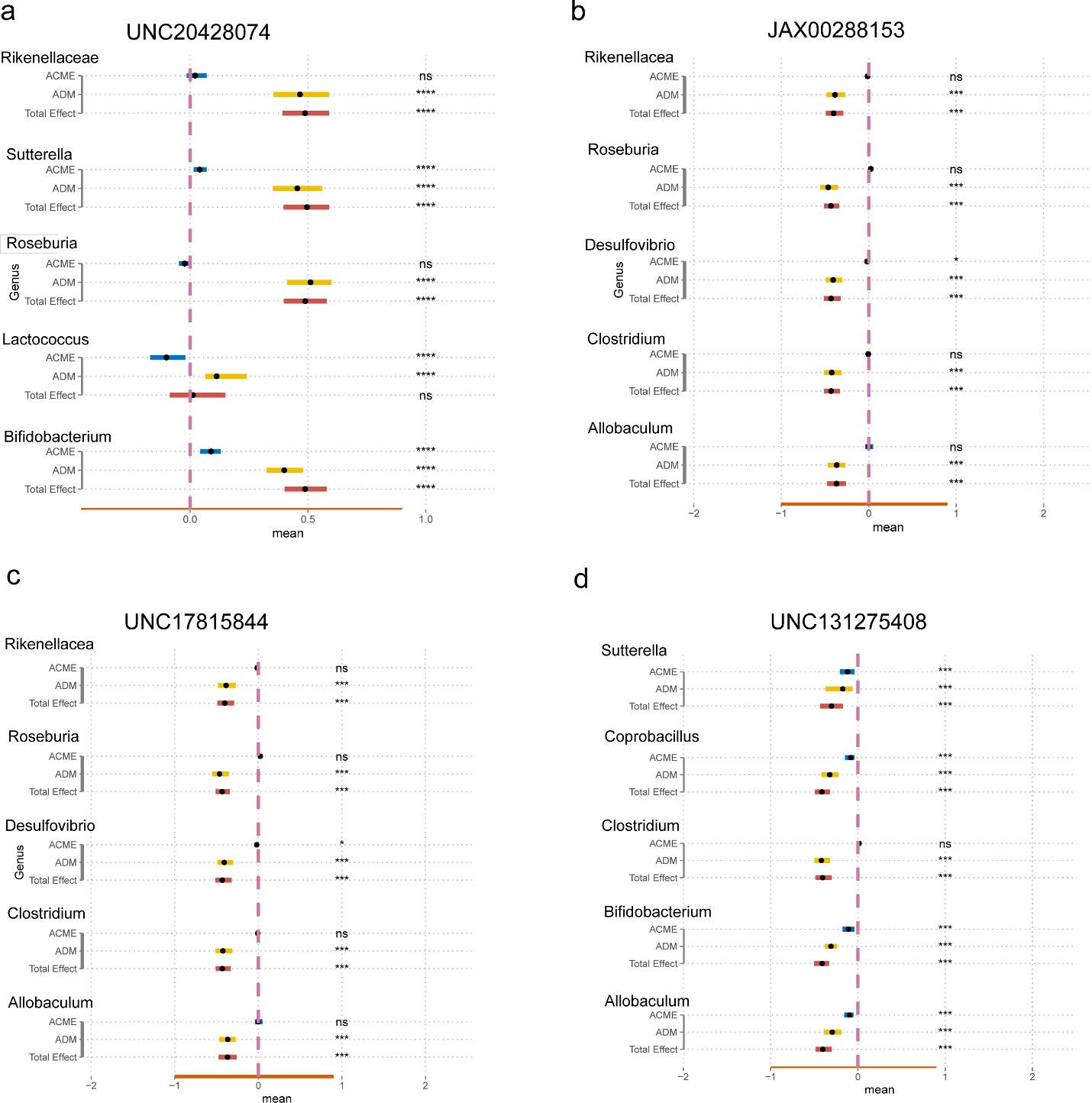


**Supplemental Figure S4** Microbes mediate the genetic effects on CTS. (a) SNP UNC20428074.

(b) SNP JAX00288153. (c) SNP UNC17815844. (d) SNP UNC131275408.


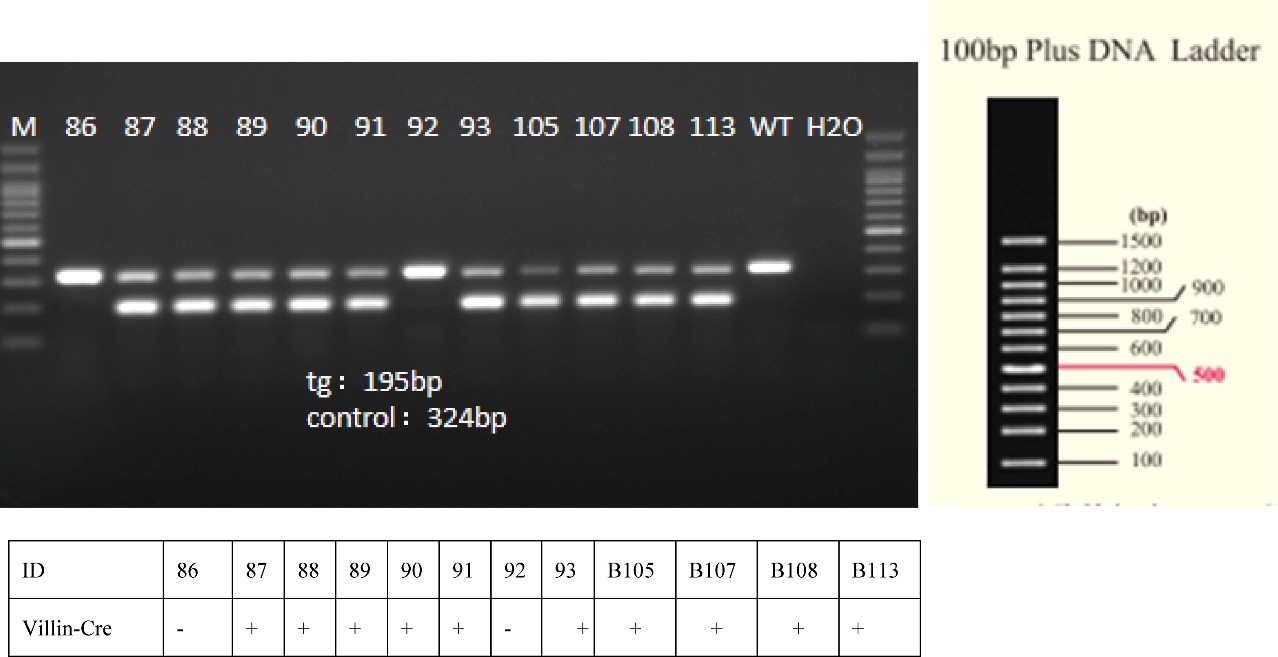


**Supplemental Figure S5** Establishment and Characterization of intestine-specific Duox2 deficient mice by polymerase chain reaction (PCR). (+) are Duox2 ^fl/fl^ Villin-Cre mice, (-) are Duox2^fl/fl^ mice.


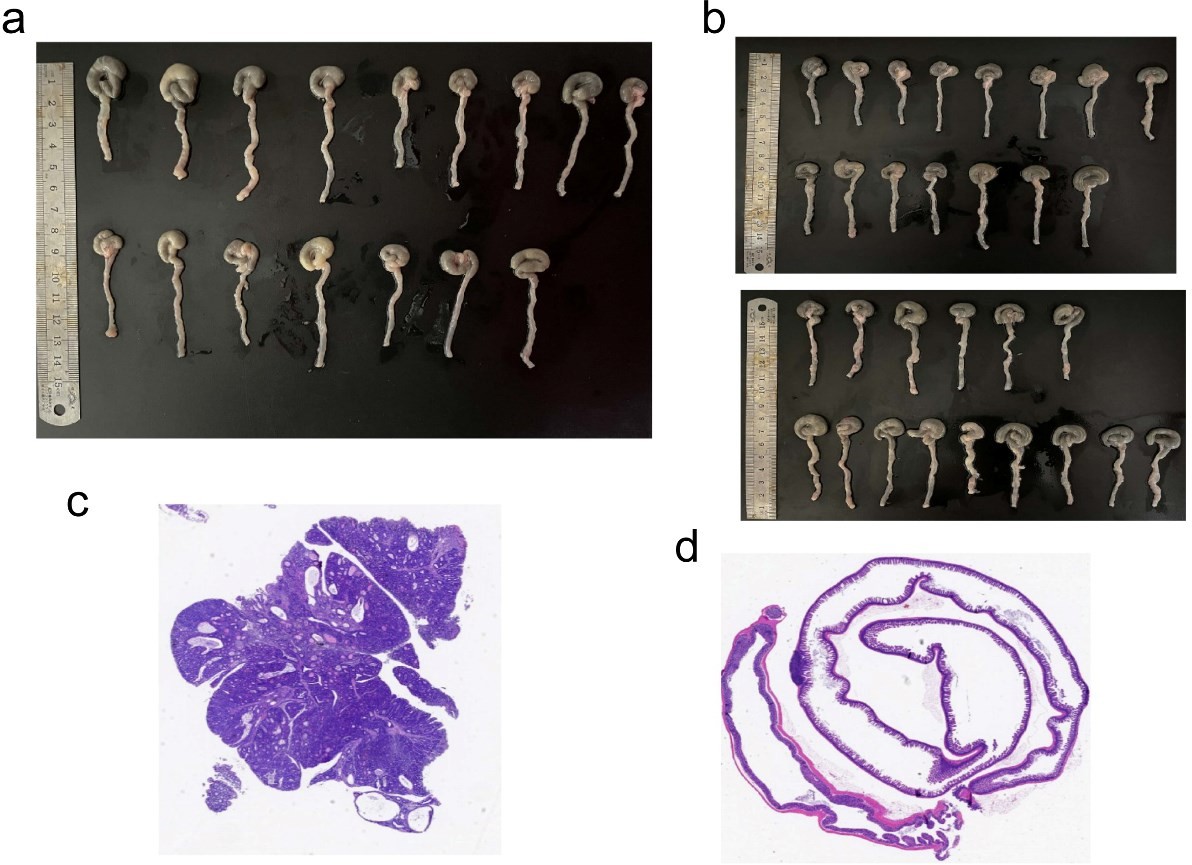


**Supplemental Figure S6** Representative photographs of colorectal tumors in WT and CKO mice.

(a) Representative images of the colon in mice from the WT mice. (b) Representative images of the colon in mice from the and CKO mice. (c) Representative H&E image of colorectal tumor showing hyperplastic glands with low-grade dysplasia. (d) Representative H&E image of colon without tumors.


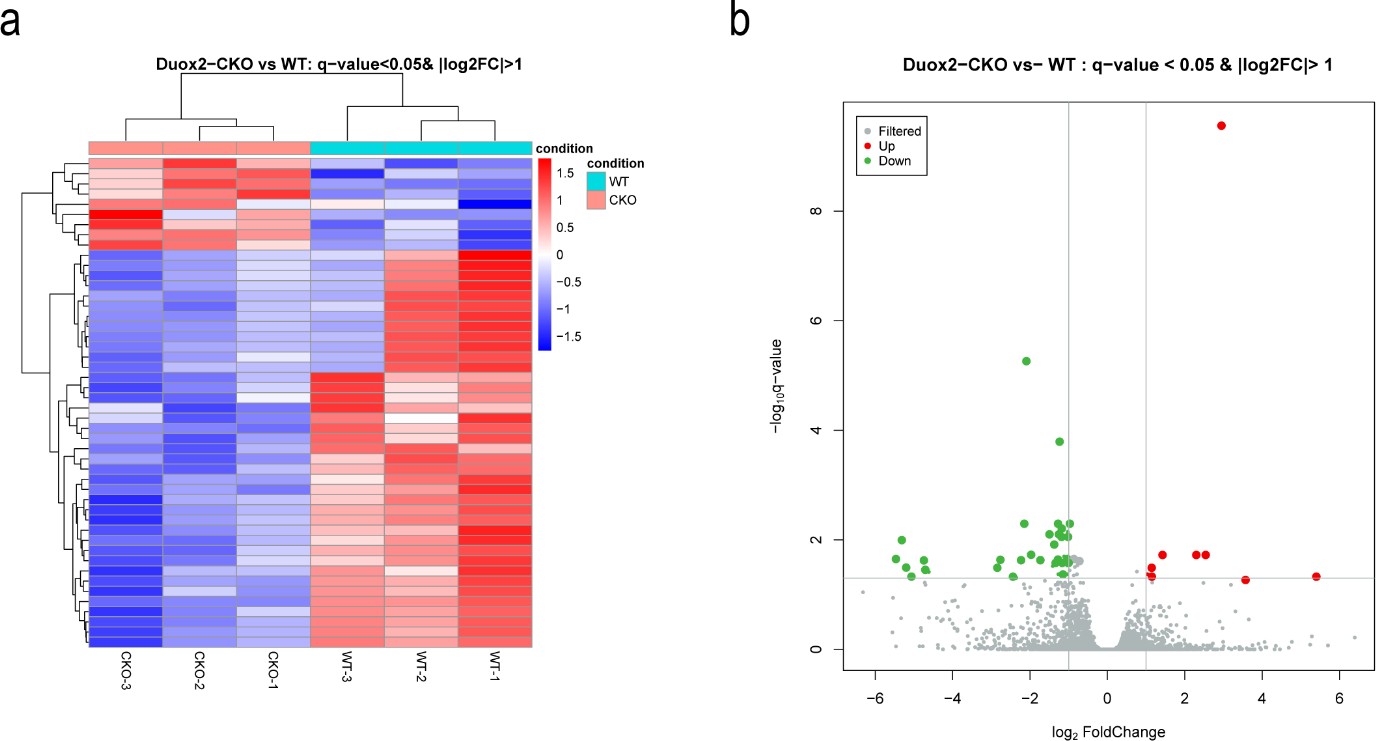


**Supplemental Figure S7** Differentially expressed genes were identified between WT samples and CKO samples. (a) Heatmap of the differentially expressed genes between WT samples and CKO samples, with red indicating higher expression and blue indicating lower expression. (b) volcano map of the differentially expressed genes between WT samples and CKO samples, with red indicating higher expression and green indicating lower expression.

# Supplemental Tables

**Supplemental Table S1** List of collaborative cross mice used in this study and tumor status in each mouse (Note: mice highlighted in red were excluded for analysis).

**Supplemental Table S2** List of SNPs and p-values obtained from the genome-wild association study of tumor susceptible phenotype.

**Supplemental Table S3** Human homolog and their alteration in colon adenocarcinoma and overlap with human GWAS. Significantly downregulated genes (FDR ≤ 0.001 and fold change ≤ 0.5) highlighted in Green and significantly upregulated genes (FDR ≤ 0.001 and fold change ≥ 2.0) highlighted in red.

**Supplemental Table S4** List of overrepresented KEGG pathways among 334 candidate susceptibility genes that significantly differentially expressed in human colon adenocarcinoma.

**Supplemental Table S5** Number of sequencing reads at genus level in each sample.

**Supplemental Table S6** List of the p-values obtained by Mann-Whitney test between low and

high colon tumor susceptibility (CTS) groups and the adjusted p-value by Bonferroni for each genus.

**Supplemental Table S7** The p-values for each SNP associated with the abundance of genus (Significant p-value <1.0E-06 is highlighted in red).

**Supplemental Table S8** List of SNPs significantly associated with the abundance of multiple genera.

**Supplemental Table S9** List of the p-values obtained by Mann-Whitney test between *Duox2* wildtype (WT) and conditional knockout (CKO) mice the adjusted p-value by Bonferroni for each genus.

**Supplemental Table S10** List of differentially expressed genes between Duox2 wildtype (WT) and conditional knockout (CKO) mice.

**Supplemental Table S11** The use of DUOX2 expression and cancer microbiome data from TCGA-COAD cohort to generate Figure 8B, C and D.
